# ALS spinal neurons show varied and reduced mtDNA gene copy numbers and increased mtDNA gene deletions

**DOI:** 10.1186/1750-1326-5-21

**Published:** 2010-05-26

**Authors:** Paula M Keeney, James P Bennett

**Affiliations:** 1 Department of Neurology, University of Virginia, Charlottesville, Virginia, USA; 2 Department of Neurology, Virginia Commonwealth University, Richmond, Virginia, USA

## Abstract

**Background:**

Spinal cord neurons of ALS patients demonstrate reduced cytochrome oxidase histochemical activity, and ALS spinal cord tissues have increased mitochondrial DNA (mtDNA) point mutations and depleted mtDNA levels. It is presently unknown whether mtDNA abnormalities are present in single human ALS neurons.

**Results:**

Using laser capture microdissection (LCM) we isolated several hundred individual anterior spinal neurons from unfixed, frozen sections of 10 ALS and 7 age-matched CTL cervical spinal cords. DNA from each individual neuron was analyzed with multiplex qPCR for ND2, CO3, and ND4, three mitochondrial DNA genes encoding respiratory proteins. Scatterplots of individual spinal neuron results showed extensive heterogeneity of mtDNA gene levels across 4-5 orders of magnitude that were much more clustered in single Purkinje neurons isolated from CTL cerebella. Plots of ratios of ND4/ND2 and CO3/ND2 showed that many but not all ALS neurons from individuals contained low ratios of these mtDNA genes, implying greater abundances of mtDNA deletions in the major arc. Single CTL cerebellar Purkinje neurons did not contain high levels of apparent mtDNA deletions observed in anterior spinal neurons.

**Conclusions:**

At the time of ALS subjects' deaths, many but not all surviving anterior neurons in their cervical spinal cords have reduced mtDNA gene levels and increased mtDNA deletion abundances that arise for unclear reasons. If these anterior spinal neuron mtDNA gene deficiencies contribute to bioenergetic impairments, reduced synaptic function and increased risk of degeneration, then introduction into mitochondria and expression of intact mtDNA, now available through use of recently developed recombinant human TFAM, may reverse the course of ALS.

## Background

Impaired mitochondrial bioenergetic function is assuming increasing prominence as a potential causal factor in neuronal dysfunction and degeneration in adult neurodegenerative diseases[1-4]. Amyotrophic lateral sclerosis is a relatively rare neurodegenerative disease of adults that presents with a clinical phenotype deriving from progressive death of upper and lower motorneurons. Spasticity, weakness and denervation-induced muscle atrophy progress over several years to loss of swallowing and ventilation function. Death ensues unless artificial ventilation is undertaken.

90-95% of ALS occurs sporadically (sALS) and is frequently associated with translocation into neuronal cytoplasm and aggregation of TDP-43, a normally nuclear localized protein involved in RNA binding and processing [5-9]. sALS overlaps clinically and pathologically with frontotemporal lobar degeneration [6,7,10,11], where caspase-processed TDP-43 fragments are found in cytoplasmic aggregates and appear to be the major toxic species [12-14].

Surviving spinal motorneurons in ALS cases exhibit substantial loss within their populations of histochemical activity of cytochrome oxidase (CO) [15], the terminal respiratory enzyme complex in the mitochondrial electron transport chain (ETC) that is composed of three subunits coded by mitochondrial DNA (mtDNA), ten subunits coded by nuclear DNA and utilizes critical assembly factors also coded by nuclear genes [16]. Mutations of CO subunit genes in mtDNA, nDNA or CO assembly factors can depress CO activity [16-18], which is selectively reduced in ALS spinal cord homogenates [19]. ALS spinal cord tissue samples also have increased mtDNA point mutations and depressed mtDNA levels [20], both of which could contribute to reduced bioenergetic function observed in ALS motorneurons [15].

Insights into mitochondrial respiratory impairments in ALS spinal cord neurons and homogenates can be gleamed from complementary studies in Parkinson's disease. CO histochemically-negative substantia nigra neurons from aged individuals and Parkinson's disease subjects show increased abundances of mtDNA deletions that can encompass CO and complex I genes and are individually unique at their break point sequences [21,22]. It is unclear both how those deletions arise [23,24] and to what degree they contribute to neurodegeneration initiation and progression. Because magnetic resonance spectroscopy studies of living PD patients at early disease stages show reduced basal ganglia ATP levels [25], mitochondrial impairments may contribute significantly to early pathogenesis.

In the present study we measured mtDNA gene levels in individual ALS anterior spinal neurons isolated by laser capture microdissection (LCM). With multiplex qPCR we determined copy numbers of ND2, CO3 and ND4, three mtDNA genes representative of genes frequently present in major arc deletions (CO3, ND4) and normally deleted to much lesser degrees (ND2). We also carried out identical assays in extracts from CTL cerebellar Purkinje neurons as a control for the techniques used when applied to readily recognized neurons from a population not considered vulnerable in ALS.

## Results

### mtDNA ND2 gene copy numbers are equivalent in ALS and CTL spinal neurons

**Figure 1:**
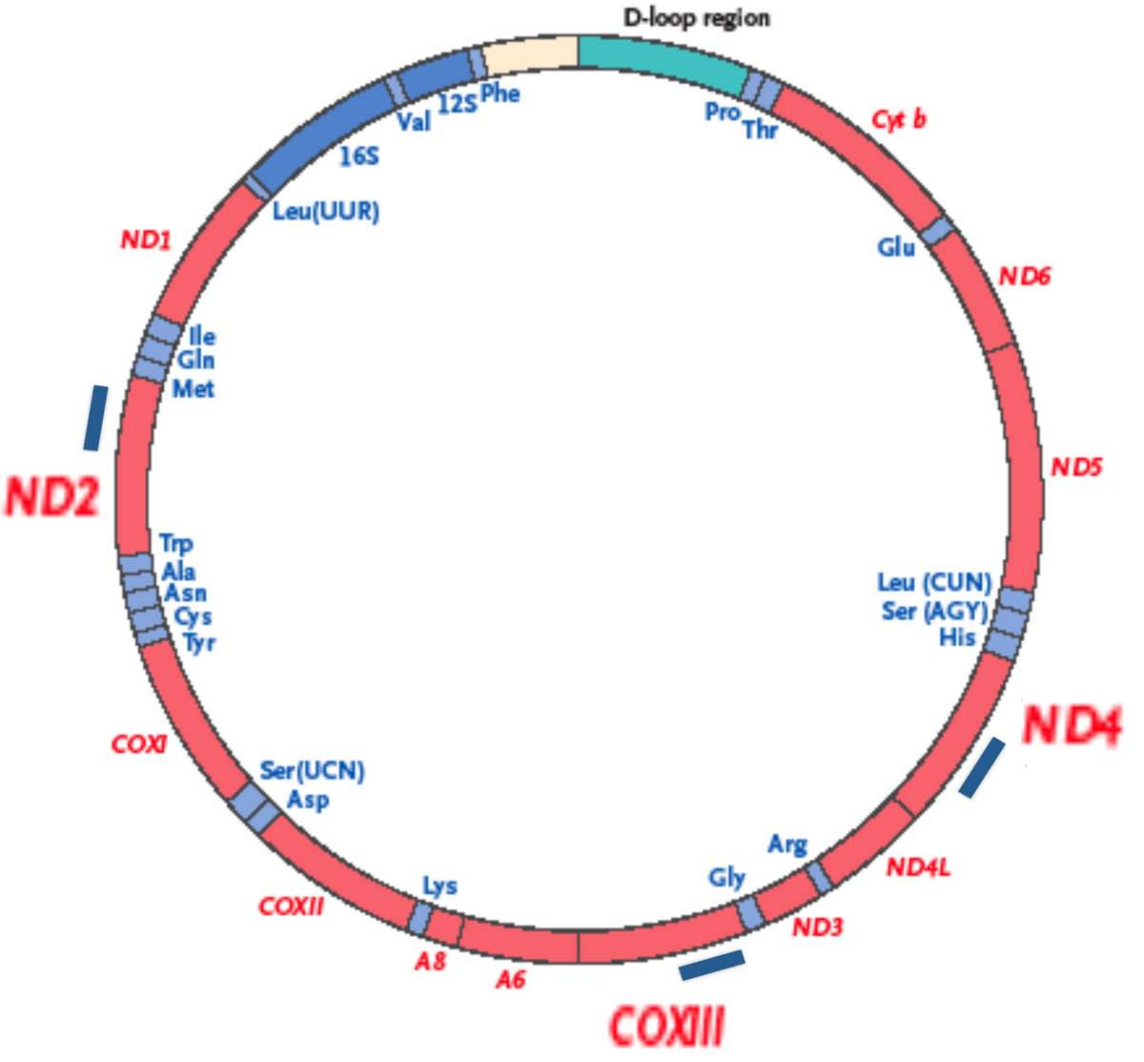
**Approximate location of the PCR products used for qPCR analysis of mtDNA genes ND2, CO3 and ND4**.

**Figure 2:**
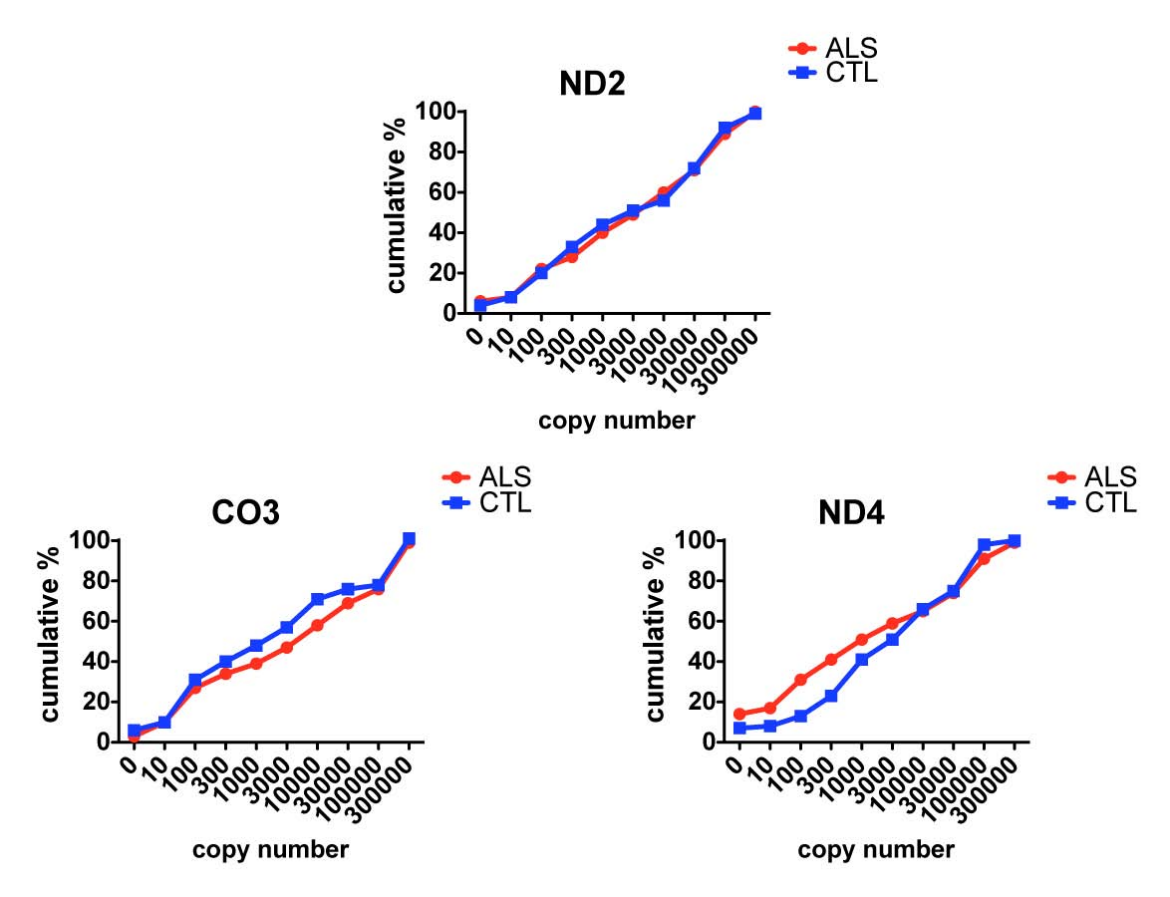
**Cumulative percentages of copy numbers for ND2, CO3 and ND4 genes in individual anterior spinal neurons from the ALS and CTL populations**.

Figure 1A shows approximate locations of PCR products generated in our qPCR assay. Figure 2A shows the cumulative percentage distributions of copy numbers for the mtDNA genes ND2, CO3 and ND4 in the ALS (n = 216 neurons) and CTL (n = 163 neurons) groups. Copy numbers were determined per neuron, independently of its size. To create the cumulative analysis graphs of Figure 2A, we plotted histograms for the percentage distributions of each gene across the gene copy number ranges shown on the x-axes. We then determined cumulative percent distribution over each copy number interval by adding each percent distribution to the sum of those preceding it.

In this population-based analysis there were no apparent differences in ND2 copy numbers between spinal neurons isolated from CTL compared to ALS neurons. For CO3, the ALS population had reduced cumulative percentages over a broad range of copy numbers. For ND4, the ALS population had higher cumulative percentages for the very low copy numbers. Figure 2A also shows that neurons from both groups possessed very broad ranges of mtDNA gene copy numbers, spanning many orders of magnitude.

**Figure 3:**
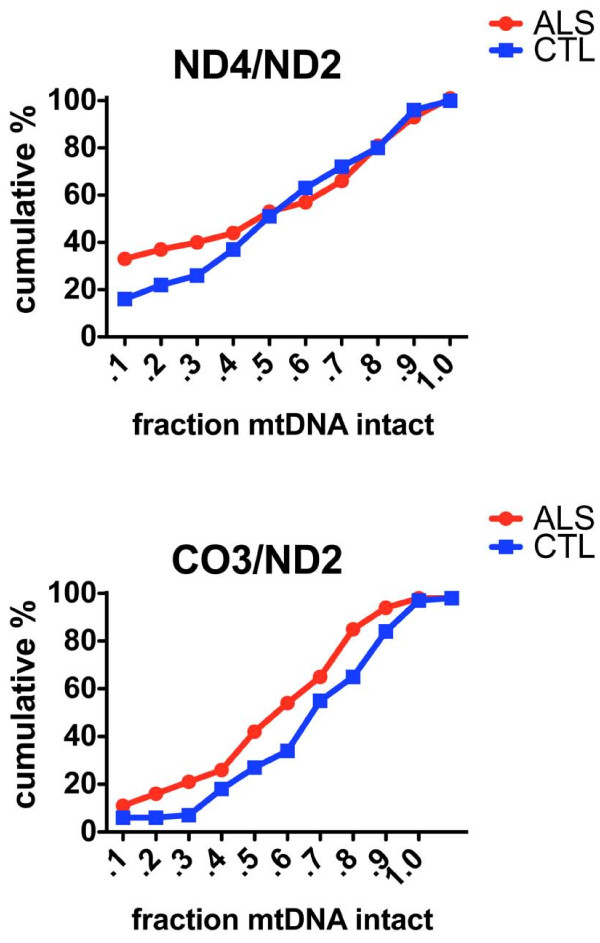
**Cumulative percentages for ratios of ND4/ND2 and CO3/ND2, estimating relative abundances of deletions in ND4 and CO3, respectively, in individual anterior spinal neurons from the ALS and CTL populations**.

**Figure 4:**
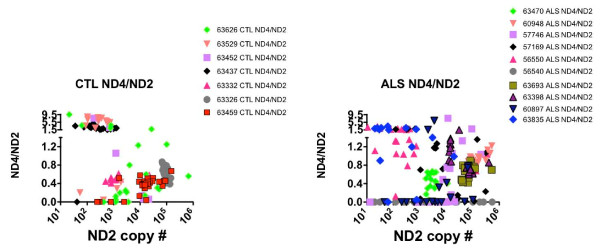
**Scatterplots relating the ND2 copy numbers to ratios of ND4/ND2 in the CTL (left) and ALS (right) individual anterior spinal neurons**.

**Figure 5:**
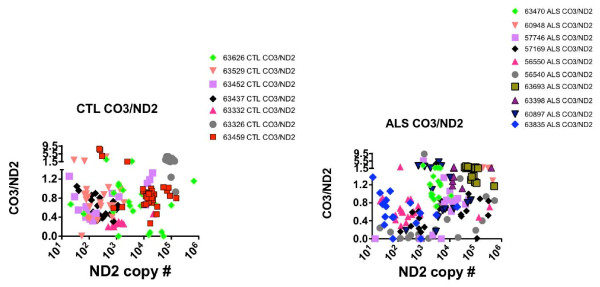
**Scatterplots relating the ND2 copy numbers to ratios of CO3/ND2 in the CTL (left) and ALS (right) individual anterior spinal neurons**.

**Figure 6:**
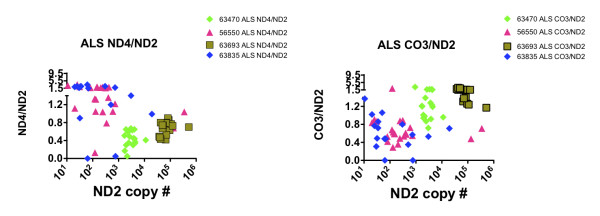
**Scatterplots showing how individual ALS cases display relatively greater deletion abundance for CO3 compared to ND4 (63835, 56550) or for ND4 compared to CO3 (63470, 63693)**.

**Figure 7:**
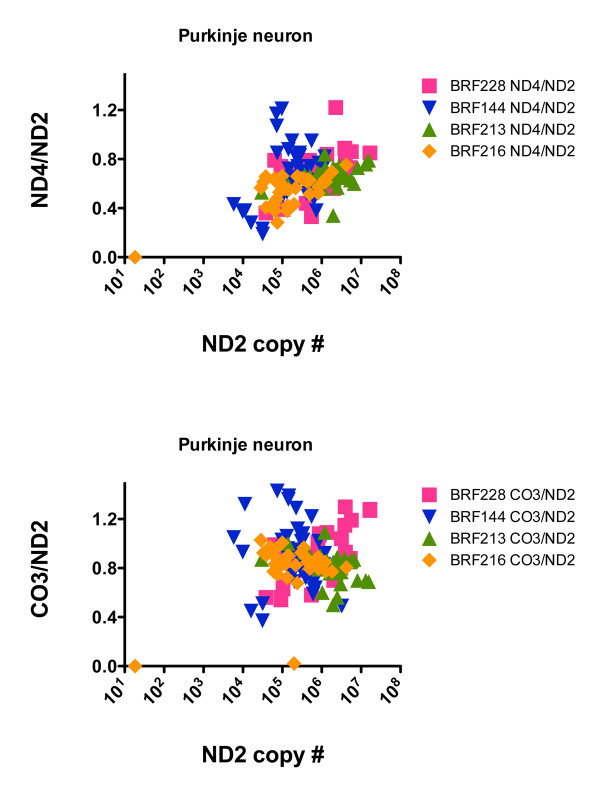
**Scatterplots relating the ND2 copy numbers to ratios of ND4/ND2 (top) and CO3/ND2 (bottom) in cerebellar Purkinje neurons from four CTL cases**.

Data presentations as in Figures 2A and 3A have the simplicity of displaying results for both populations studied (ALS, CTL), but they have the drawback of not showing any details of distributions within each sample. These details of each sample are revealed in the plots of Figures 4A, 5A, 6A and 7A. Additional File [Supplementary-material S1] provides the data for each sample's individual neurons' ND2 copy numbers, and ND4/ND2 and CO3/ND2 ratios.

### mtDNA gene deletions are increased in ALS spinal neurons

To estimate relative levels of mtDNA gene deletions we used each neuron's level of ND2 as a marker for overall mtDNA abundance, since deletions in the minor arc region are less common than those involving the major arc. We expressed ratios of CO3/ND2 and ND4/ND2 as estimates of degree of deletion involving the major arc region where each of these genes (CO3, ND4) is located. If all individual deletions present involved both ND4 and CO3, then their abundances should equal each other. However, that was not the case.

Figure 3A shows cumulative percentage plots for ND4/ND2 and CO3/ND2 on the y-axes and fraction intact mtDNA on the x-axes. In both graphs the ALS group overall had greater apparent deletion abundance, but with different distributions. For ND4 in the ALS group there was an approximate doubling of deletions present at very high abundance (80-90%), whereas for CO3 the ALS population showed a consistent increase in all deleted species between 20-80% abundance.

### mtDNA copy numbers and deletion abundances are very heterogeneously distributed

In Figures 4A and 5A the distributions of ND4/ND2 and CO3/ND2 ratios for each neuron are shown as a function of the individual levels of ND2 in each spinal cord sample from the CTL and ALS populations. Figures 4A and 5A demonstrate the extensive heterogeneity of mtDNA gene levels observed across five orders of magnitude in all spinal cord samples, the clustering (or lack thereof) among neurons from individual spinal cords, and the relationships across spinal cord samples between ND2 copy numbers and deletion abundances involving ND4 compared to CO3.

For apparent deletions involving ND4 (Figure 4A), samples with high deletion abundances (i.e, ND4/ND2 ratios < 0.2) were distributed in CTL samples across ~2 orders of magnitude of ND2 levels, but were both much more abundant and distributed across ~5 orders of magnitude of ND2 levels in the ALS population.

For apparent deletions involving CO3 (Figure 5A), samples with high deletion abundance (CO3/ND2 ratios < 0.2) were relatively rare in the CTL group but more abundant in the ALS group, where they tended to cluster in the midrange of ND2 copy number levels.

### mtDNA deletions in ALS spinal neurons can be greater in either ND4 or CO3 and appear to relate to ND2 copy number levels

Figure 6A shows data extracted from Figures 4A and 5A for selected spinal cord samples of the ALS population. For two samples (63693, 63470), relative deletion abundances were greater for ND4 compared to CO3, whereas for two other samples (56550, 63835) the opposite was the case. Also, for the two samples with higher apparent levels of deletions involving ND4 compared to CO3, their copy number levels were orders of magnitude higher than for the two samples where deletions involving CO3 appeared more abundant than those involving ND4.

### ND2 copy numbers and apparent deletions involving ND4 and CO3 are different in Purkinje neurons

We applied the same approach to cerebellar Purkinje neurons isolated from four disease-free CTL cases. Figure 7A shows that the distributions in 147 Purkinje neurons of ND2 copy numbers and levels of apparent deletions involving ND4 or CO3 were much more restricted than those found in anterior spinal neurons from either the ALS or CTL cases. In Purkinje neurons, apparent deletions involving ND4 tended to cluster in the 20-40% range, and those involving CO3 in the 10-30% range.

## Discussion

In this study we used multiplex qPCR to measure the levels of three mtDNA protein coding genes in anterior spinal neurons from cervical cord samples of ALS patients or age-matched CTL's. Using levels of ND2 as a reference for mtDNA copy number, we found wide variations across five orders of magnitude in the samples. For some subjects, levels of ND2 in individual neurons tended to cluster together, whereas for other subjects there was greater spread in ND2 levels and little apparent clustering. In the ALS population, cases where many neurons contained very high deletion abundances tended to have the most widespread distributions of ND2 copy numbers. Identical analyses of single Purkinje neurons from four CTL cases suggested that our techniques were not likely sources of artifacts to explain our observations.

Our findings suggest that anterior spinal neurons from older persons show widespread distributions of mtDNA gene copies, whereas such diversity is less apparent in Purkinje neurons. To our knowledge, ours is the first study to quantitate copy numbers of multiple individual mtDNA genes in isolated single neurons from human postmortem specimens. mtDNA is replicated using a RNA-primed DNA polymerase gamma [26-28], and the control of mtDNA levels is believed to reside in a multitranscription factor "mitochondrial biogenesis" program [29-31]. It is not clear why anterior spinal neurons would show such diversity of mtDNA gene levels, which for many samples spanned several orders of magnitude and for others were very tightly clustered. Our findings indicate that study of mitochondrial transcription factors' gene expressions in these spinal neurons, particularly PGC-1α, NRF1, NRF2 and PRC [29,30,32] might be helpful in understanding the wide variations in mtDNA gene levels observed.

In the ALS samples we found both greater abundances of apparent deletions involving ND4 and CO3 and variations across spinal cord samples as to which gene was more deleted. mtDNA gene deletions have been reported to increase with aging in human substantia nigra neurons and become dominant in samples obtained from persons >80 years old at time of death [21,22]. Our mean age for the ALS specimens was 63 years, and we observed no relationship between apparent deletion abundance and subject age. The three ALS specimens with the greatest numbers of very high ND4 deletion levels were from individuals who died at 50, 61 and 62 years of age.

The origins of mtDNA deletions may involve replication errors induced by repair of oxidatively damaged bases [23,24]. Substantia nigra neuron mtDNA deletions are characterized by an abundance of direct repeat sequences at the deletion break points [21-23], suggesting that DNA polymerase gamma may "skip" to similar sequences if single strand breaks generated during repair of oxidative damage persist during mtDNA replication.In our study we did not attempt sequencing of mtDNA deletion breakpoints, so we cannot propose whether a similar mechanism might be operative in anterior spinal neurons.

Our findings suggest that in individual ALS anterior spinal neurons from some but not all subjects, and to a lesser extent in age-matched CTL spinal neurons, very high abundance mtDNA deletions exist that would be predicted to produce impaired respiratory capacity, similar to what has been observed in individual substantia nigra neurons [21,22]. It is not clear if this situation is causal for anterior spinal neuron death in ALS, but the severity of the anticipated respiratory deficit would be both substantial and markedly reduce capacity for synaptic activity. Such neurons would also be predicted to have decreased capacity to modulate swings in cytosolic calcium levels and survive excitotoxic stresses.

Our findings support the concept that some spinal neurons die in ALS because of accumulation of high abundance mtDNA deletions that are predicted to eliminate meaningful mitochondrial ATP production. Although it is not clear how these mtDNA deletions arise, effective therapy would involve supplementation of intact mtDNA to mitochondria *in vivo *[33].

For treating ALS, where many neurons show complete loss of detectable ND4 or CO3, it will likely be necessary to provide to and express intact mtDNA in motorneurons. However, it is important to note that based on our findings, such an approach might not be effective for all ALS subjects. Some of our cases showed abundant mtDNA copy numbers and relatively low levels of deletions. This observation speaks to the heterogeneity of a complex disease such as ALS, and we do not propose accumulation of mtDNA deletions as a universal cause of synaptic failure or neurodegeneration in this condition.

Future studies need to address how such apparent mtDNA deletions arise, whether there are failures in the mitochondrial biogenesis program in aging neurons, and if so, do they arise from genetic, epigenetic or post-translational causes (or some combination). From a therapeutic development perspective, it is necessary to show that rhTFAM can deliver and express intact mtDNA to anterior spinal neurons and that mitochondrial respiratory capacity in those neurons is increased.

## Conclusions

Using multiplex qPCR we measured levels of ND2, CO3 and ND4, three mtDNA genes that code for essential respiratory proteins, in individual human anterior spinal neurons laser captured from cervical spinal cords of age-matched ALS and CTL subjects. We found a widespread distribution of mtDNA gene levels that could not be accounted for solely by the techniques used. Estimated mtDNA deletions involving either ND4 or CO3 genes were increased in the ALS group, involved either ND4 or CO3 to a greater extent in each subject, and were not always predictable based on mtDNA copy numbers. Although the origins of such deletions are not clear, those of very high abundance are predicted to impair respiration and may increase vulnerability to neurodegeneration. Introduction of intact mtDNA to these neurons is potentially therapeutic but likely would not improve spinal neurons with low deletion abundance and non-depressed mtDNA copy numbers.

## Methods

### Tissue and Laser Capture Microdissection

Frozen specimens of cervical spinal cord from six neurologically normal, one case without motorneuron disease but felt to have Alzheimer's dementia, and ten ALS patients were obtained from the National Disease Research Interchange, Philadelphia, PA http://www.ndriresource.org. The clinical features of these cases are listed in Additional File [Supplementary-material S2] Table S1.Ten micron frozen sections of unfixed spinal cord were placed on uncoated glass microscope slides and stored at -80°C. In preparation for laser capturing, slides were air dried 2 min. at room temperature, stained for 7 min in 1% methylene blue (Electron Microscopy Sciences, Hatfield, PA), dehydrated for 2 min. each in 70 and 95% ethanol, 4 min each in 100% ethanol and xylenes and held in a desiccated chamber. Single motor neurons in the anterior grey matter were dissected using an Arcturus PixCell IIe system (Molecular Devices, Sunnyvale, CA).

### DNA Extraction and Real-Time Quantitative PCR

The DNA from each neuron was extracted using an overnight incubation at 65°C in 50 ul TK buffer (0.5 M Tris-HCl, pH 8.9, 20 mM EDTA, 10mM NaCl, 5% (v/v) Tween 20 and 2 mg/ml Proteinase K).Extracted samples were diluted 1:2 with sterile TE buffer and used as the template in multiplex qPCR reactions targeting mitochondrial genes. Sequences of primers and probes are listed in Additional File [Supplementary-material S3], Table S2.qPCR was carried out with human mtDNA copy number standards in quadruplicate 25 ul volumes using BioRad iQ Multiplex Power Mix and an iCycler with an iQ5 detection system (BioRad, Hercules, CA). The cycling protocol details were:activation at 95°C for 5 min. followed by 50 cycles of 95°C melting for 10 sec. and 50° annealing/extension for 1 min.The copy number standards were generated by PCR amplification of the entire human mitochondrial genome using primers located at the BamH1 restriction site in ND6. The isolated PCR product was a single band of the appropriate ~16 kbase size. Data was analyzed using the iQ5 software and standard curves generated from the copy number standard.

## Abbreviations

ALS: amyotrophic lateral sclerosis; LCM: laser capture microdissection; mtDNA: mitochondrial DNA; rhTFAM: recombinant human mitochondrial transcription factor A.

## Competing interests

The authors declare that they have no competing interests.

## Authors' contributions

PMK and JPB designed experiments; PMK carried out experiments and analyzed data; JPB wrote the paper. Both authors read and approved the final manuscript.

## Supplementary Material

Additional file 1 Data on each samples individual neurons' ND2 copy numbers, and ND4/ND2 and CO3/ND2 ratios. Click here for file

Additional file 2 Clinical Features of Spinal Cord Cases Click here for file

Additional file 3 Sequences of probes and primers for human mtDNA genes. Click here for file
