# Supplementary material for: ALS spinal neurons show varied and reduced mtDNA gene copy numbers and increased mtDNA gene deletions
Source: Mol Neurodegener. 2010 May 26;5:21. doi: 10.1186/1750-1326-5-21 (PMC2889994; doi:10.1186/1750-1326-5-21)
Supplement: Additional file 2 — Clinical Features of Spinal Cord Cases [file 1750-1326-5-21-S2.DOC]

**Supplemental Table 1: Clinical Features of Spinal Cord Cases**

**PMI**

**Case Sex Age (hours) Medical History_________________________________**

| **Control:** |  |  |  |  |  |
| --- | --- | --- | --- | --- | --- |
| **63326** | 54 | M | 17.0 |  | recurrent sz, GERD, collapsed lung, ruptured spleen |
|  |  |  |  |  | 2 d. on ventilator, death from seizure |
| **63332** | 80 | M | 24.0 |  | 5 yr hx of AD, prostate Ca, HTN, osteoporosis, neck fusion, |
|  |  |  |  |  | tobacco use, colycystectomy, hernia repair, death from prostate Ca |
| **63437** | 76 | M | 8.0 |  | CMP, CHF, HTN, sleep apnea and unspecified dementia, |
|  |  |  |  |  | death from cardiac arrest |
| **63452** | 66 | F | 19.0 |  | esophageal Ca, diabetes, 1 ppd tobacco use, cause of death CVA |
| **63529** | 67 | F | 24.0 |  | 8 mo hx of Pancreatic Ca, mets to liver, Ja, dementia related to Ja |
|  |  |  |  |  | death from pancreatic Ca |
| **63626** | 66 | M | 17.0 |  | CHF, CAD, HTN, HL, peptic ulcer, sleep apnea, death from CHF |
| **64539** | 75 | M | 24.0 |  | aneurysm, stents in 2008, NIDDM, Sarcoidosis, back Sx, flu shot, |
|  |  |  |  |  | varicose veins - no Sx, cataract Sx, death from cardiac arrest |
| **ALS:** |  |  |  |  |  |
| **56540** | 50 | F | 9.0 |  | 5 hx of ALS, c-sections, lasik & wrist Sx |
| **56650** | 64 | F | 6.3 |  | 8 yr hx of ALS, GERD, hepatitis, gallbladder & uterus removed, |
|  |  |  |  |  | 5 yr s/p feeding tube |
| **57169** | 80 | F | 16.0 |  | 4 yr hx of ALS, MD, CHF, HTN, PE, back Sx, 4 yr s/p pneurmonia, |
|  |  |  |  |  | 5yr s/p TIA |
| **57746** | 61 | M | 11.8 |  | 3 wk dx of ALS, CABG, HTN, CAD, GERD, alcohol & tobacco use, |
|  |  |  |  |  | knee replacement, bleeding ulcer, 3 d. on ventilator |
| **60498** | 48 | M | 17.0 |  | 3 yr hx of ALS, cyst on feet, coughing & fever 3 wk prior to death |
| **60897** | 62 | F | 10.0 |  | ALS, 4 yr s/p appendectomy, bilateral conjunctivitis |
| **63398** | 70 | M | 21.0 |  | 7 mo hx of ALS, alcohol use, HTN, sepsis, GERD, pneumonia, |
|  |  |  |  |  | rheumatic fever as a child, death from ALS |
| **63470** | 67 | M | 8.0 |  | 1 mo hx of ALS, NIDDM, MD, alcohol & tobacco use, corotid |
|  |  |  |  |  | endodectomy, angioplasty, prostate hx, feeding tube, |
|  |  |  |  |  | cause of death pneumonia |
| **63693** | 75 | F | 16.0 |  | ALS, bleeding ulcer 2 yrs ago, varicose veins, stroke, death from ALS |
| **63835** | 57 | M | 11.0 |  | 8 mo hx of familial ALS, Gene: A4GF01G, HL, alcohol use, |
|  |  |  |  |  | death from ALS |

**PMI**, post mortem interval, **M**, male; **F**, female**; CABG**, coronary artery bypass graft; **CAD**, coronary artery disease; **HTN** hypertension; **mets,** metastases; **HL**, hyperlipidemia; **GERD**, gastoesophageal reflux disease; **Sz**, seizures; **CHF**, congestive heart failure; **CMP**, cardiomyopathy; **Ca**, cancer; **AD**, Alzheimer’s disease; **MD**, macular degeneration, **Sx,** surgery; **PE**, pulmonary embolism**; Ja**, jaundice
