# Supplementary material for: ALS spinal neurons show varied and reduced mtDNA gene copy numbers and increased mtDNA gene deletions
Source: Mol Neurodegener. 2010 May 26;5:21. doi: 10.1186/1750-1326-5-21 (PMC2889994; doi:10.1186/1750-1326-5-21)
Supplement: Additional file 3 — Sequences of probes and primers for human mtDNA genes. [file 1750-1326-5-21-S3.DOC]

| **Oligo Name** | **Sequence 5' to 3'** | **Modification** |
| --- | --- | --- |
| ND2 probe | CACGCAAGCAACCGCATCCATAAT | 5'-FAM; 3'-BHQ1 |
| ND2 sense | AAGCTGCCATCAAGTATTTCC |  |
| ND2 antisense | GTAGTATTGGTTATGGTTCATTGTC | |
|  |  |  |
| COX3 probe | CGAAGCCGCCGCCTGATACTG | 5'-TET; 3'-BHQ1 |
| COX3 sense | TTTCACTTTACATCCAAACATCAC |  |
| COX3 antisense | CAATAGATGGAGACATACAGAAATAG | |
|  |  |  |
| ND4 probe | AGCCAGAACGCCTGAACGCAG | 5'-TAMRA; 3'-BHQ2 |
| ND4 sense | TGGCTATCATCACCCGATG |  |
| ND4 antisense | TGAGTAGTAGAATGTTTAGTGAGC |  |
|  |  |  |

**Supplemental Table 2. Sequences of probes and primers for human mtDNA genes.**
